# Supplementary material for: Content-rich biological network constructed by mining PubMed abstracts
Source: BMC Bioinformatics. 2004 Oct 8;5:147. doi: 10.1186/1471-2105-5-147 (PMC528731; doi:10.1186/1471-2105-5-147)
Supplement: Additional File 2 — The original results of the above study (non-essential files are deleted to keep the file size under the limit set by BMC bioinformatics). [file 1471-2105-5-147-S2.bz2 › chilibotAdditionalFile2/dip20/513ID10344732E10815/html/CCND1_AR.html]

 


 **CCND1** and **AR** 
  
Found 122 abstracts in PubMed, retrieved 20.  
 

 What does Google say? 
 PDF only 
| .edu only 

---

**Interactive relationship** (e.g. stimulation, inhibition, etc)

**Stimulatory relationship**- Finally, microarray analyses were performed that showed increased expression of Bcl  [ **CCND1** ]  3, an NFkappaB IkappaB factor, was correlated with AIS  [ **AR** ] .  Ref: 12538159 Ann N Y Acad Sci, 2002
- In contrast,  **AR** , a specific ErbB1 ligand, induced sustained ERK1 2 and Elk1 phosphorylation, increased cyclin D1  [ **CCND1** ]  mRNA and protein, and increased DNA synthesis activity.  Ref: 12869389 Circ ResCirc Res, 2002
- Down regulation of bcl  [ **CCND1** ]  2 protein and mRNA by DHT was inhibited by coincubation with the antiandrogen bicalutamide, an agent that competitively inhibits binding of DHT to androgen receptor  [ **AR** ] .  Ref: 12629413 J Urol, 2003
- Treatment of LY  **ar**  cells with the MEK inhibitors PD 98059, U0126, and PD 184352 led to a loss of phosphorylated ERK1 and ERK2, a reversal of nuclear p50 homodimer DNA binding, and a decrease in Bcl  [ **CCND1** ]  2 protein expression.  Ref: 12801933 J Biol Chem< MedlineTA>J Biol Chem, 2003
**Neutral relationship**- ... a sustained ERK Elk1 activation could be the major signaling cascade for  **AR**  induced upregulation of cyclin D1  [ **CCND1** ]  protein in HTASMCs.  Ref: 12869389 Circ ResCirc Res, 2003
- In addition to the effects on cell proliferation, FGF2 maintains the survival of  **AR**  PC3 clones through a positive modulation of the Bcl  [ **CCND1** ]  2 protein and down modulates  **AR**  protein expression, allowing the escape of selected clones from androgen regulation.  Ref: 12430142 Prostate, 2002
- CONCLUSION In the presence of an active  **AR** , the combined production of FGF2 and FGF BP may play an important role in the progression of prostate cancer through the selection of  **AR**  clones expressing high levels of Bcl  [ **CCND1** ]  2.  Ref: 12430142 Prostate, 2002
- Pretreatment with PD98059 suppressed  **AR**  induced cyclin D1  [ **CCND1** ]  mRNA and protein.  Ref: 12869389 Circ ResCirc Res, 2002

**Non-interactive relationship** (e.g. studied together, co-existance, homology, etc.)

- Flow cytometric stains for antiapoptotic Bcl  [ **CCND1** ]  2 and Bcl  [ **CCND1** ]  xL showed that neither factor was well correlated with AIS  [ **AR** ] , although both were increased transiently upon T cell activation.  Ref: 12538159 Ann N Y Acad Sci, 2002
- To investigate the mechanism of the AI growth displayed by the GOF p53 mutants, Western blotting or ELISA were used to examine the expression of the androgen receptor  [ **AR** ]   **AR** , the  **AR**  regulated prostate specific antigen PSA, as well as Akt and Bcl  [ **CCND1** ]  2 under androgen depleted conditions.  Ref: 12727844 Cancer Res, 2003
- We examined the impact of beta  **AR**  blockade with metoprolol on myocardial remodeling, apoptosis, pro apoptotic Fas, Fas ligand, Bax, and Bcl  [ **CCND1** ]  X S and anti apoptotic Bcl  [ **CCND1** ]  X L and Bcl  [ **CCND1** ]  2 gene expression, and Bcl  [ **CCND1** ]  X L and Bcl  [ **CCND1** ]  X S protein in post infarction HF in rats.  Ref: 12738230 J Mol Cell Cardiol, 2003
- In a previously published report, we described the NF kappaB status for two murine B cell lymphoma cell lines, LY as apoptosis sensitive and LY  **ar**  apoptosis refractory, and provided evidence that NF kappaB1 p50 homodimers contribute to the expression of Bcl  [ **CCND1** ]  2 in the LY  **ar**  line.  Ref: 12801933 J Biol Chem< MedlineTA>J Biol Chem, 2003
- The status of antisense targeting of genes relevant to prostate cancer, including bcl  [ **CCND1** ]  2, bcl  [ **CCND1** ]  xL, clusterin, androgen receptor  [ **AR** ]   **AR**  and IGFBPs, are reviewed.  Ref: 12400997 Cancer Metastasis Rev, 2002
- We conclude that adrenergic activation, such as occurs in HF, increases pro apoptotic Bcl  [ **CCND1** ]  X S expression via the beta 1  **AR** . beta  **AR**  blockade in HF reduces myocardial apoptosis.  Ref: 12738230 J Mol Cell Cardiol, 2003
- In contrast, expression of differentiation specific proteins prostate specific antigen and androgen receptor  [ **AR** ]  or proliferative regulatory proteins c myc, cyclin D1  [ **CCND1** ] , p27 were down regulated by hypoxia.  Ref: 12481256 Prostate, 2003
- The status of antisense targeting of several genes, including bcl  [ **CCND1** ]  2, bcl  [ **CCND1** ]  xL, clusterin, androgen receptor  [ **AR** ]  and IGFBPs, relevant to prostate and other cancers, are reviewed.  Ref: 12643471 Curr Drug Targets, 2003
